# Dietary Effects of Fasting on the Lipid Panel

**DOI:** 10.2174/011573403X257173231222042846

**Published:** 2024-02-23

**Authors:** Jason Hourizadeh, Rezwan Munshi, Roman Zeltser, Amgad N. Makaryus

**Affiliations:** 1 Department of Internal Medicine, St. John’s Riverside Hospital, Yonkers, NY, USA;; 2 Department of Cardiology, Nassau University Medical Center, East Meadow, NY, USA;; 3 Department of Cardiology, Donald and Barbara Zucker School of Medicine at Hofstra/Northwell Health, Manhasset, NY, USA

**Keywords:** Cholesterol, lipids, fasting, diet, time-restricted eating, caloric restriction

## Abstract

**Introduction:**

Dietary habits, such as the Mediterranean diet and the Dietary Approaches to Stop Hypertension (DASH), have been shown to improve cardiac health. Another more recent popular form of dieting incorporates periods of fasting known as intermittent fasting. The two main forms are alternate-day fasting and time-restricted eating.

**Methods:**

PubMed search and literature review was undertaken. This review evaluates the current literature regarding the effects of the fasting dietary model and other types of fasting upon the lipid panel.

**Results:**

There have been studies that have shown that intermittent fasting does provide a benefit in cardiovascular health, weight loss, and hypertension. However, the effect on cholesterol and triglyceride levels during intermittent fasting is in question.

**Conclusion:**

The effect that fasting has on one’s lipid panel is unclear, there are studies that show that different forms of fasting affect the lipid panel in various ways. There are studies that show that intermittent fasting does improve one’s lipid profile and provides health benefits. Randomized controlled clinical trials with a large sample size are needed to evaluate the effects that intermittent fasting has based on race, ethnicity, gender, obesity, dyslipidemia, diabetic and healthy patients, and will lead to definitive evidence of lipid panel outcomes beyond current evidence based solely upon observational cohorts with numerous and multifactorial confounding factors and biases.

## 1 INTRODUCTION

Between 2015-2018, in adults 20 years old or older in the United States, about 94 million people (38.1%) had a total cholesterol level higher than 200 mg/dL, roughly 28 million (11.5%) had a total cholesterol (TC) level greater than 240 mg/dL, and 68 million (27.8%) had a low-density lipoprotein cholesterol (LDL-C) level greater than 130 mg/dL [1]. During the years 2009 – 2016, about 7.1% of youth (6-19 years old) had an elevated total cholesterol level [1]. Having hypercholesterolemia increases the risk of heart disease and stroke, both of which are leading causes of death in the United States [2]. Various treatment options for hypercholesterolemia include dieting, exercise, and medication. In an analysis performed in 2016, the U.S. estimated healthcare spending for patients diagnosed with hyperlipidemia was $26.4 billion [1]. The U.S. population has gained interest in dieting and weight loss programs in order to improve health. Some of these diet plans have limited data to prove their efficacy. Each diet focuses on the foods one consumes with the goal of losing weight. However, each diet can alter one’s lipid profile differently. There are many health benefits associated with weight loss such as reducing hypertension and atherosclerotic cardiovascular disease risk, and preventing type 2 diabetes mellitus, cancer, and obesity [3, 4].

The American Heart Association recommends that a person’s diet must consist of vegetables, fruits, whole grains,legumes, poultry, fish, and nuts to help decrease the LDL-C, which will thereby reduce cardiovascular and all-cause mortality [5]. Popular diets utilized to reduce cholesterol levels are therapeutic lifestyle changes (created by the National Institute of Health's National Cholesterol Education Program), vegetarian diets, and vegan diets. Intermittent fasting has gathered interest due to the popular belief that it will help decrease cholesterol and triglyceride (TG) levels, as well as result in weight loss and maintenance of that weight loss. Prior studies such as the study conducted by Meng *et al.* [6] suggest that intermittent fasting and energy-restricted diets improve circulating TC, LDL-C and TG concentrations [6].

## 2 MATERIALS AND METHODS

**Fig. (1):**
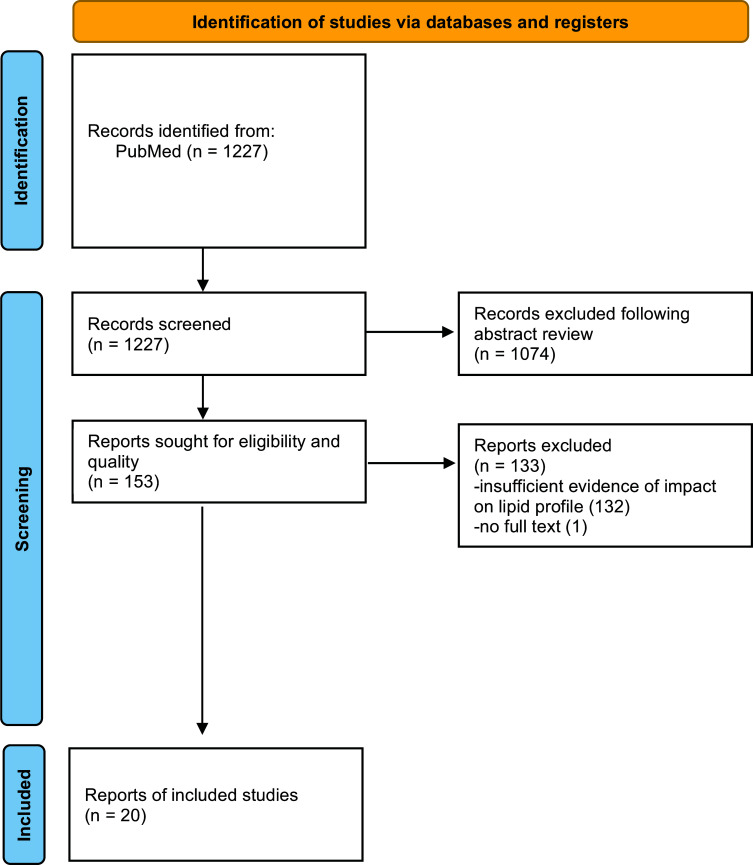
PRISMA flowchart diagram.

We searched PubMed using the following terms: (Intermittent fasting) AND (alternate day fasting OR ADF) AND (lipid profile OR lipid panel). Inclusion criteria consisted of studies evaluating patients undergoing a period of fasting and reporting of the participant's lipid profile before and after the fast. Multiple searches were conducted during the period of June 21^st^, 2021, through August 27^th^, 2022. This review explores the outcomes and effects on the lipid panel that the various forms of fasting exert (Fig. **1**).

## 3 TYPES OF FASTING AND NOTED OVERALL EFFECTS UPON THE LIPID PANEL

### 3.1 Intermittent Fasting

**Fig. (2):**
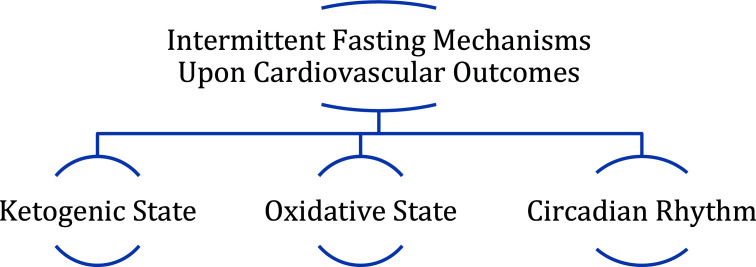
Proposed mechanisms of benefit from intermittent fasting.

Intermittent fasting utilizes the concept of restricting food intake, meaning limiting the time period when one consumes food. It is similar to caloric restriction in that both diets are focusing on restricting food intake, however caloric restriction’s aim is to decrease one’s calorie intake throughout the day [7]. The three proposed mechanisms of how intermittent fasting can provide benefit and improve cardiovascular outcomes are the oxidative stress hypothesis, circadian rhythm theory, and the ketogenic state (Fig. **2**) [7].

The oxidative stress hypothesis elucidates that decreased energy intake causes the mitochondria to produce less free radicals leading to less inflammation [7]. Second, the circadian rhythm theory states that syncing eating periods to one’s organ’s circadian rhythm will optimize glucose and fat utilization. Third, Intermittent fasting induces a ketogenic state, which has been linked to reduction in cardiovascular risk factors such as a decrease in blood pressure and adipose tissue [7]. In a ketogenic state, one utilizes fatty acids and ketones for energy rather than glucose, this phenomenon is called intermittent metabolic switching. Also, a ketogenic diet promotes weight loss because processing ketones utilizes more energy when compared to glucose [7].

**Fig. (3):**
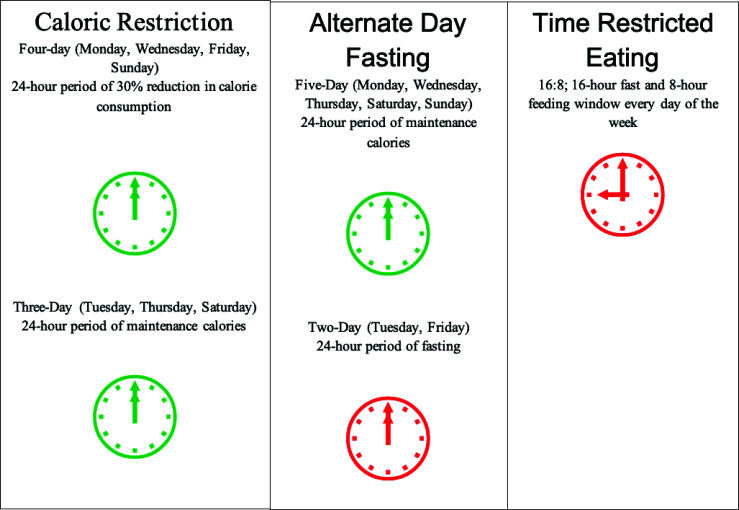
Different forms of intermittent fasting (right columns) compared to caloric restriction (left column).

Intermittent fasting consists of two different regimens (Fig. **3**), time-restricted eating and alternate fasting [7]. Time-restricted eating focuses on the hours one may eat during the day; some variations can be a 16-hours fast with 8-hours eating times or a 20-hours fast with 4-hours eating times [7]. Alternate day fasting (ADF) may consist of a 24-hours fast followed by a 24-hours period of non-restricted eating which can be done multiple times a week such as a 5:2 schedule where there are 2 fasting days mixed in with 5 non-restrictive eating days [7]. Although decreased caloric intake may occur during intermittent fasting, this is not the intended mode of action. Other forms of fasting are caloric restriction and dietary restriction [8]. Caloric restriction is the reduction of kilocalories by a certain percentage, and dietary restriction is the reduction of one or more components of the diet with minimal to no reduction in total kilocalories [8]. These differences highlight and are felt to contribute to the expected positive effects of intermittent fasting over plain caloric or dietary restriction. However, Stote *et al*. studied the effect of consuming only one meal per day (fasting about 20 hours per day) in healthy, normal-weight adults and it was found that total cholesterol, LDL and HDL were all elevated [9].

### 3.2 Religious Fasting

While religious fasts are primarily upheld for spiritual purposes, they have the potential to have an effect on physical health. Religious fasting can include everything from limiting meat intake to more stringent dietary modifications. Three religious fasting periods are those of Orthodox Christianity (Nativity, Lent, and the Assumption), the Daniel Fast and Islamic Ramadan. In the Nativity fast, which is 40 days long, the individual fasting abstains from eggs, meat and dairy products [8]. On Wednesdays and Fridays, the individuals fasting do not eat olive oil and fish. During Lent, which is a 48-day period, those fasting abstain from eggs, meat and dairy products, along with olive oil on weekdays and fish every day except for March 25^th^ and Palm Sunday. During Assumption, a 15 day fast, those fasting abstain from dairy products, eggs and meat [8]. Outside of these fasting periods, every Wednesday and Friday one abstains from eating cheese, eggs, milk, fish, meat and olive oil [6]. Therefore, overall dietary consumption is restricted for about 180-200 days a year [8]. During the Daniel fast, refined foods, white flour, preservatives, additives, sweeteners, flavorings, caffeine, and alcohol are forbidden [8]. This is most commonly a 21-day fast observed in January, but it can be observed at any time of the year [8].

Ramadan is a Muslim religious fast that takes place during the ninth month of the Islamic calendar and lasts between 28 and 30 days [8]. Ramadan is a period of fasting that restricts one from eating, drinking and smoking from sunrise to sunset [8]. The consumption of food and drink is unrestricted during the hours of darkness. There are two meals which many Muslims take part in during Ramadan: a larger meal after sunset and a lighter meal before dawn. Therefore, Ramadan is similar to intermittent fasting, as both incorporate periods of fasting and feasting. An important difference between intermittent fasting and Ramadan is that during intermittent fasting, fluid intake is permitted during the fast period, while during Ramadan it is prohibited [8]. As both intermittent fasting and Ramadan are similar types of fasting, they also have similar effects on a patient’s lipid profile. Hassanein *et al*. assessed diabetic patient’s lipid profiles before and after Ramadan and the data showed that TC, TG, HDL, and LDL all worsened, rather than being improved [9, 10].

### 3.3 Binge Eating Disorder

Binge-eating disorder is recurrent episodes of binge eating with marked distress, and episodes occurring at a minimum of once a week for three months [11]. An episode of binge eating can be defined as the following: Eating an amount of food that is definitely larger than what most people would be able to in a certain amount of time (usually less than two hours) or having a lack of control while eating [11]. The binge-eating episodes will be accompanied with at least three of the following: Eating more rapidly than normal, eating until feeling uncomfortably full, eating an excessive amount of food when not feeling hungry, or eating alone because of the feeling of embarrassment with the amount one is eating [11].

### 3.4 Eating Disorders such as Anorexia and Bulimia – Binge and Purge

Anorexia nervosa is the restriction of energy intake relative to requirements, leading to low body weight and the intense fear of becoming fat or gaining weight [11]. There are two subtypes of anorexia nervosa: One is binge-eating, defined as engaging in recurrent episodes of purging behavior (self-induced vomiting, misuse of laxatives, enemas or diuretics) or restricting-type where one loses weight primarily by excessive exercise, dieting and fasting [11]. However, bulimia nervosa is recurrent episodes of binge eating at least once a week for three months [11].

Since patients with anorexia nervosa with binge/purging type are in a constant state of fasting, their lipid panel may be affected by this. In a study conducted by Giovinazzo *et al*., it was shown that this population of patients had an elevated total cholesterol, LDL, HDL and apolipoprotein A1 [12].

### 3.5 Starvation: The Ultimate form of Fasting

Starvation is a severe deficiency in the caloric intake required for the maintenance of life. During the early period of human starvation, humans initially undergo glycogenolysis, the breakdown of glycogen and gluconeogenesis, for the formation of glucose [13]. After about 2-3 days of fasting, the human body undergoes lipolysis in order to break down lipid stores to form fatty acids and ketone bodies to be used as the primary source of fuel [13]. Ketogenesis continues as long as starvation continues and eventually leads to tissue breakdown and eventual cessation of organ function leading to death.

## 4 MECHANISMS OF EFFECT UPON THE LIPID PROFILE DURING FASTING

**Fig. (4):**
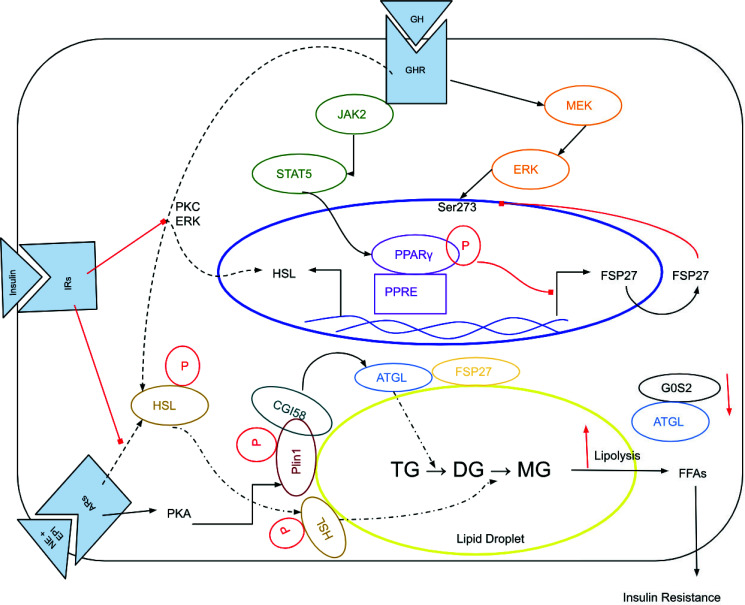
Mechanisms of the effect of fasting upon the lipid profile at the molecular level [107].

In states of fasting, the levels of growth hormone (GH) increases, which induces lipolysis and insulin resistance that can have negative effects on cholesterol and triglyceride levels (Fig. **4**). GH activates the MEK-ERK pathway, which phosphorylates PPARγ at Ser273, inactivating it and downregulating FSP27 [14]. This decrease in FSP27 expression causes increased lipolysis and higher circulatory free fatty acids (FFAs) and insulin resistance [14]. Furthermore, GH induces lipolysis by activating hormone-sensitive lipase (HSL) *via* PKC and ERK [14]. Adrenergic signaling *via* adrenergic receptors can also stimulate lipolysis by activating the lipolytic cascade, which involves adipose triglyceride lipase, HSL and monoacylglycerol lipase [14]. However, insulin can inhibit lipolysis by suppressing GH-induced PKC, ERK activity, and the adrenergic pathway [14]. One possible mechanism that GH causes insulin resistance is that GH leads to elevated FFA levels and suppresses pyruvate dehydrogenase. This shows that there is substrate competition between glucose and lipid intermediates at the starting point of the citric acid cycle [14].

Lipoproteins can be divided into groups based on their apolipoproteins, lipid composition and function [15]. Apolipoprotein B (ApoB) is a structural protein that is an important part of chylomicrons, very low-density lipoproteins (VLDL), intermediate density lipoproteins (IDL) and low-density lipoprotein particles (LDL) [15]. As VLDL delivers triglycerides to tissues, it is lipolyzed into IDL and then LDL particles [15]. LDL-cholesterol can then be taken up by cells that need exogenous cholesterol, although most LDL is cleared *via* the liver [15].

On the other hand, high density lipoprotein (HDL), does not contain ApoB. HDL removes excess cholesterol from peripheral tissues and then returns them to the liver for clearance from the body [15]. Insulin plays a major role in coordinating lipoprotein metabolism. During the anabolic state, high insulin levels act on adipocytes to promote triglyceride uptake and inhibit FFA release, storing the FFA for later use [15]. In a fasting state, this process is reversed; a drop in insulin levels causes FFA to be secreted from adipocytes and delivered to the liver [15]. The liver then forms VLDL from the FFAs, and secretes it into the bloodstream [15]. In the absence of insulin, adipocytes are unable to uptake triglycerides, causing other tissues, such as skeletal muscle and liver to utilize it [15].

The LDL receptor (LDLR), which binds to ApoB100 and ApoE, plays a central role in serum cholesterol levels. Insulin increases LDLR-mRNA *via* transcription factor SREBP-1c [16]. Proprotein convertase subtilisin/kexin type 9 (PCSK9) is a secreted protein that binds to LDLR and promotes its breakdown [15]. Humans with gain in function mutations of PCSK9 have increased levels of LDL-cholesterol compared to humans with loss of function mutations in PCSK9 [15]. Therefore, it is possible that the ability of insulin to induce PCSK9 can reduce LDLR expression, thereby not allowing LDL to be stored in the liver and remain in the bloodstream [15].

Anorexia nervosa is accompanied by endocrine dysregulation, including hypothalamic-pituitary dysregulation, which is seen in chronic starvation [16]. Anorexia nervosa is a state of acquired growth hormone (GH) resistance caused by chronic lack of nutrition leading to an increased GH secretion but decreased systemic insulin-like growth factor 1 (IGF1) [16]. Elevated GH is able to maintain euglycemia *via* gluconeogenesis and lipolysis, in states of chronic starvation [16]. A mechanism of GH resistance and the resultant low circulating levels of IGF1 is in states of fasting where fibroblast growth factor 21 (FGF21) is elevated and is believed to inhibit STAT-5, a mediator of intracellular GH effects [16]. Thus, plasma FGF21 levels are inversely related with plasma levels of IGF-1 [16]. Furthermore, low levels of insulin, which is seen in states of undernutrition, downregulate hepatic GH receptor expression [16]. Elevated GH levels from the negative feedback mechanism of the hypothalamic and pituitary gland is caused by low levels of IGF-1 [16].

Many believe that intermittent fasting or alternate day fasting can have a positive effect on the serum lipid profile. However, the opposing belief is that these forms of dieting can worsen the lipid profile through increases in GH leading to increased lipolysis and release of FFA [17]. Nevertheless, once the fast is broken and feeding has been restarted, the effect of GH ends [17]. Resuming feeding leads to increased insulin production. It has been shown that insulin increases hepatic LDL receptor gene expression and LDL receptor binding. During periods of fasting, decreased insulin can lead to decreased LDL receptor expression in the liver causing increased LDL circulation in the blood [17]. Therefore, periods of fasting can lead to elevated serum LDL by decreased LDL receptor expression on the liver and decreased LDL clearance by the liver [17].

## 5 LITERATURE FINDINGS RELATED TO DIFFERENT TYPES OF FASTING BASED ON THE POPULATION EVALUATED

Many studies have examined the effects of different lengths and forms of fasting and how it affects both proatherogenic and anti-atherogenic lipids. There are three components to proatherogenic lipids, which include elevated TG, elevated LDL, and decreased HDL. All three of these elements have an independent risk factor for cardiovascular disease [18]. In atherogenic dyslipidemia, impaired insulin signaling increases lipolysis, thereby increasing the conversion from TG into free fatty acids, which then increases VLDL [18]. Increased VLDL will lead to increased LDL production and a decrease in HDL [18]. The antiatherogenic lipid is HDL [19]. Its antiatherogenic property includes decreasing endothelium inflammation and oxidative stress and increases the production of nitric oxide and promoting endothelial cell survival [19]. These studies evaluate a range of patient types undergoing the various forms of fasting and it is important to consider each patient profile separately.

### 5.1 Overweight and Obese Population

Radhakishun *et al*. specifically studied the obese population, and evaluated the patients the week prior to Ramadan, the last week of Ramadan and then six weeks after the conclusion of Ramadan [20]. When comparing the labs from the week prior to Ramadan to during the fourth week of Ramadan, total cholesterol increased by 14%, LDL cholesterol rose by 21.2%, and HDL cholesterol increased by 3.7%. However, all values returned to baseline at the tenth week [20]. Comparatively, Minsuk *et al.* held a study that looked at the effects of 24 days of alternate day caloric restriction (ADCR) versus exercise plus alternate day caloric restriction (E-ADCR) for total of 24 exercise sessions and how it impacts cardiometabolic risk factors in overweight and obese adults [21]. After the eighth week of the study period, the TG, TC, and HDL in the ADCR group increased by 11%, 2.1%, and 4.4%, respectively [21].

Bonnet *et al*., conducted a systematic review and meta-analysis of randomized trials with an average length of 8.6 weeks of overweight/obese and normal weight patients studying the effects of skipping breakfast on different cardiometabolic risk factors [22]. Using the fixed-effect inverse variance approach, LDL increased (9.30 mg/dL, (*P*=0.008) and TC increased (15.54 mg/dL, *P* < 0.001) in the group that skipped breakfast compared to the group that ate breakfast [22].

A study compared ADF in obese patients. Two groups, one with ADF and low fat (LF) diet and another with ADF and high fat (HF) diet were compared to see its effects on cholesterol sub-particles such as LDL [23]. Body weight decreased (*P*<0.0001) by 4.3 ± 1.0 kg (4.8 ± 1.1%) and 3.7 ± 0.7 kg (4.2 ± 0.8%) in the ADF-HF and ADF-LF group, respectively [23]. LDL cholesterol was reduced (*P*<0.0001) by 19 ± 8 mg/dl (18 ± 5%) among ADF-HF and 28 ± 7 mg/dl (25 ± 3%) among ADF-LF [23]. The proportion of small LDL particles decreased (*P*<0.005) by 8 ± 2% and 11 ± 3% in the ADF-HF and ADF-LF groups, respectively [23]. HDL cholesterol and HDL size remained unchanged [23]. They concluded that the ADF-HF diet is equally as effective as the ADF-LF diet in improving LDL particle size and distribution (Table **1**).

### 5.2 Diabetic Population

Bener *et al*. held a crossover study where type 2 diabetic patients were evaluated at week four and week twelve of Ramadan, which showed that LDL-cholesterol increased by 8.5% [24]. However, HDL-cholesterol, TC, and TG all decreased [24]. Therefore, someone observing the month of Ramadan with underlying diabetes should be cautious as extended periods of fasting in this subpopulation can increase LDL. Khan *et al*. conducted a study assessing the effect of fasting on lipid panels in type 2 diabetic patients during Shabann (month prior to Ramadan), Ramadan, and Shawwal (six day fast after Ramadan) [25]. It showed that total cholesterol increased from 173.08 ± 36.20 mg/dl to 177.83 ± 37.58 mg/dl during Shawwal. The LDL increased by 8.2% during the month of Ramadan and increased from 112.64 ± 34.19 mg/dl to 119.0 ± 35.1 mg/dl at the Shawwal visit. However, HDL and TG both decreased [25] (Table **2**).

A study conducted by Hassanein *et al*. assessed patients with type 2 diabetes 4-6 weeks prior to Ramadan and 2-4 weeks after Ramadan [10]. The data showed that the TC, TG, HDL, LDL increased by 4.1%, 9.5%, 1.2%, and 4.2%, respectively [10]. This study showed that even 2-4 weeks post Ramadan, the patient’s lipid profile was still negatively affected [10].

### 5.3 Anorexic Population

The DSM-V definition of anorexia nervosa is restriction of energy intake relative to requirements, leading to a significantly low body weight in the context of age, sex, developmental trajectory, and physical health [11].

A study was conducted that compared three groups of patients, one with anorexia nervosa with binge/purging type (AN-B), bulimia nervosa, and a control group. After a 12-hour overnight fast, in the AN-B group, the TG was 1.50 ± 1.06 mmol/L (*P*<0.05), VLDL-cholesterol was 0.12 ± 0.04 mmol/L, IDL-cholesterol was 0.69 ± 0.26 mmol/L, LDL-cholesterol was 3.40 ± 0.75 mmol/L (*P*<0.05) and HDL-cholesterol was 1.49 ± 0.26 mmol/L; all values were elevated compared to the bulimia nervosa group [26]. Comparatively, a systematic review held by Giovinazzo *et al.*, showed that anorexic patients with binge/purging type have elevated total cholesterol, LDL, HDL and apolipoprotein A1 [12].

Weinberger *et al*. held a cross sectional study with fifty-eight anorexic women compared to fifty-eight control group women to assess the metabolism of lipoproteins in patients with anorexia nervosa [27]. The results showed that the HDL, total cholesterol, and LDL were 7%, 8.9% and 11.9% higher in the AN compared to the control group, respectively (Table **3**) [27].

In all the studies that the anorexic patient population was studied, the analysis of the lipid panel proved that the LDL and total cholesterol were consistently elevated. This can be explained in anorexic patients due to elevated lipolysis, decreased cholesterol removal, and greater activity of cholesteryl-ester-transfer protein activity [12].

### 5.4 Healthy Population

Ziaee conducted a cohort study with eighty-one students from the Tehran University of Medical Sciences during the month of Ramadan and evaluated the triglyceride, cholesterol, LDL, HDL and VLDL before and after Ramadan [28]. Results showed that glucose and HDL decreased, while LDL increased by 3.8% during the month of Ramadan [28]. However, during this time there was no significant change in total cholesterol, TG and VLDL [28].

Stote *et al*. conducted a randomized crossover study with two treatment periods 8 weeks in length. The effects of eating one meal per day (fasting about 20 hours per day) versus consuming all calories in three meals in healthy, normal weight adults was studied [9]. Upon examining the lipid panel, the total cholesterol, LDL and HDL were all elevated by 11.7%, 16.8%, and 8.4%, respectively, in patients who consumed one meal per day compared to those who ate three meals per day [9].

Lars Savendahl *et al*. studied the effect of acute starvation (total of 7 days), on serum lipids in healthy, non-obese patients [17]. In response to one week of fasting, the patient’s total cholesterol, LDL and apolipoprotein B increased by 37.3%, 66.1% and 65.0%, respectively [17]. This increase was associated with weight loss [17]. Interestingly, a larger increase in serum cholesterol was seen in patients with a lower body weight compared to those with a higher body weight [13]. The data showed that there was no effect on TG and HDL [17].

Horne *et al*. conducted a systematic review of randomized clinical trials of the effects of intermittent fasting [29]. One of the trials was the Fasting and Enhanced Expression of Longevity Genes during Food Abstinence (FEELGOOD), where a Latin-square crossover design was used to examine a 24-hours period of fasting and a 24-hours period of eating [29]. During the fasting period, it showed temporary increased levels in total cholesterol, LDL, HDL, and decreased triglycerides. Horne conducted two other studies which reported similar data [29-31].

Another study conducted by Horne *et al* assessed lipid profile in healthy patients undergoing short-term fasting, defined as one-day water-only fasting, followed by a 24-hours period of eating. After the one-day fast, there was an increase in TC (4.2%), LDL and HDL (5.5%) [32]. However, after refeeding, these same participants’ parameters returned to baseline [32]. This shows that fasting even one day can alter the lipid profile; however, it can revert to baseline after one resumes eating.

Mirmiran *et al*., held a systematic review and meta-analysis studying the effects of fasting during the month of Ramadan and the effects it has on the lipid profile [33]. They found no significant effect on circulating TG, TC, and LDL-C levels [33]. HDL-C and VLDL-C significantly decreased after fasting [33]. A significant increase in LDL-C was observed in athletic subjects and healthy subjects [33]. TG changes were associated with age, baseline TG values, and weight change during the fasting period [33].

Akaberi *et al*. conducted a prospective observational study, assessing a patient's lipid profile during the month of Ramadan. TC, HDL, LDL increased by 4.5%, 28.4%, and 5.5%, respectively. However, TG improved after Ramadan [34].

A study held by Lamine *et al*., looked at the lipid profile of healthy students three weeks before Ramadan, the fourth week of Ramadan and three weeks after the end of Ramadan [35]. They found that the TC, HDL, and LDL worsened by 13.5%, 18.2% and 21.7% respectively, however three weeks after Ramadan the values returned to baseline [35]. Many of these studies show that fasting can worsen one's lipid profile, but once eating is resumed, the levels return to baseline (Table **4**).

When evaluating the effects that Ramadan fasting has on serum lipid levels, it is important to assess the nutritional diet, patient’s medical history and biochemical response to starvation. Diabetic patients, in comparison to obese patients, may have different responses to fasting, which can be found in the studies of Bener and Radhakishun. Commonly, the articles studying the fasting effects during Ramadan all showed an increase in LDL-cholesterol, while other parameters remained the same or decreased. When comparing the anorexic patient population to the participants being studied during Ramadan, it seems that the changes in the lipid profile depended on whether the patients are diabetic or obese.

## 6 RESULTS AND DISCUSSION

### 6.1 Summary of Effects of Fasting upon the Lipid Panel

#### 6.1.1 Overall Trends and Similarities

Based on these findings, intermittent fasting, whether incorporating time restricted eating or alternate day fasting, will influence a person’s lipid panel. These articles show that whether healthy, diabetic or obese, fasting can worsen one’s lipid profile– most commonly elevating one’s total cholesterol and LDL-cholesterol. However, a review article by de Cabo *et al* regarding the effects of intermittent fasting on health states that intermittent fasting has benefits for many health conditions including obesity, diabetes mellitus, cardiovascular disease, neurologic disorders, and cancers [36]. The article also states that the most effective regimen is either restricting one's eating time to a 6-8-hours window or using the 5:2 technique [36]. Furthermore, the effect upon the lipid panel is determined by the diet followed by the patient, and once the dieting is discontinued, the lipid profile often normalizes as soon as one day [32].

#### 6.1.2 Type of Diet Employed along with Fasting Greatly Influences Lipid Profile Outcomes

Upon starting an intermittent fasting diet, it is imperative to take note that the food one consumes will influence overall health and more specifically, the lipid profile [37]. A ketogenic diet includes consuming a very low carbohydrate and high fat content diet, which induces the liver to produce ketone body B-hydroxybutyrate, a metabolic energy source, which can be used by the brain as an energy source when there are low levels of carbohydrates [37]. Common foods in the keto-diet are coconut oil, butter, eggs, avocados, cheese, and meat [37]. Therefore, undertaking a ketogenic diet with intermittent fasting can negatively affect the lipid panel because one’s diet is centered around different types of fats, which will ultimately increase cholesterol [37].

Both the Mediterranean diet and the Dietary Approaches to Stop Hypertension (DASH) diet are used to help lower one’s cholesterol [38]. These diets consist of vegetables, fruits, low-fat or fat free dairy products, whole grains, lean protein sources, seeds, nuts, and liquid vegetable oils [38]. Therefore, if one were to use the Mediterranean diet or the DASH diet in combination with intermittent fasting, this will reduce the risk of an elevated lipid profile.

#### 6.1.3 Intermittent Fasting and Weight Loss

A study was conducted in order to evaluate the effects of time-restricted eating (TRE) on weight loss [37]. They placed patients in two groups, either caloric restriction (CR) (1200-1500 kcal for women, 1500-1800 kcal for men) alone or caloric restriction plus TRE, meaning that one can only eat for eight hours a day from 8:00 a.m. to 4:00 p.m [39]. It was found that even though the TRE group had a greater weight loss compared to the CR group, it was not statistically significant [39]. The study concluded that among obese patients, TRE was not found to reduce body fat or metabolic risk factors or increase weight loss when compared to the CR group [39]. However, this study may have some potential flaws, the initial one being that the study was underpowered, meaning there were not enough participants to show the difference in weight [40]. Although they were not statistically significant, the following outcomes were all improved in the TRE group compared to the CR group: Waist circumference, body fat mass, body fat percentage and visceral fat [40]. Another potential issue is that TRE and CR were being treated as independent variables; however, they are most likely dependent variables as many people eat large portions and eat too often [40]. Therefore, by reducing the hours in the day that one eats, the caloric intake is reduced without deliberate calorie counting. For this reason, one cannot conclude that TRE has no additional benefits [38]. Another study was conducted to assess the association between meal intervals and weight trajectory in adults. They tracked the time from the first meal to the last meal and found that time-restricted eating was not a useful strategy for long-term weight loss [41].

A study tested whether the timing of food intake has an effect on maintaining weight loss and body composition in patients who recently went through a period of weight loss [42]. They studied patients over an 18-months period, and patients were assigned into the following four groups: Those considered regular morning breakfast eaters (breakfast 5-7 days a week), irregular to rare early morning breakfast eaters (breakfast 0-4 days a week), avoidance to rare post-dinner evening eating (evening snacks 0-2 times a week), and frequent to habitual post-dinner evening eating (evening snacks 3-7 times a week) [42]. It was concluded that if patients ate breakfast regularly (5-7 days a week), they would regain on average 2.95 kg of body weight on average, which is 0.59 kg lower than in people who ate breakfast intermittently (0-4 days a week) [42]. Also, if participants consumed only a post-dinner snack 0-2 times per week, they would gain 0.83 kg less than those who ate post-dinner snacks 3-7 times per week [42]. What can be learned from this is when one is undertaking intermittent fasting, specifically time-restricted eating, one must determine which hours to eat and which to fast correctly. This is supported by the study by Elahy *et al.*, showing that eating breakfast consistently and decreasing post-dinner snacking will increase weight loss.

## CONCLUSION

The effect that fasting has on one’s lipid panel is unclear, there are studies that show that different forms of fasting affect the lipid panel in various ways. There are also variables that can further impact lipid profiles, including past medical history, body composition and the length of the fasting. These studies show that intermittent fasting and fasting in general does not necessarily improve one's lipid profile, but can rather worsen it. There are studies that show that intermittent fasting does improve one’s lipid profile and provides health benefits. Further research needs to be done to interpret the relationship between fasting and lipid profiles, and how long these effects last. Randomized controlled clinical trials with a large sample size are needed to evaluate the effects that intermittent fasting has based on race, ethnicity, gender, obesity, dyslipidemia, diabetic and healthy patients, and will lead to definitive evidence of lipid panel outcomes beyond current evidence based solely upon observational cohorts with numerous and multifactorial confounding factors and biases.

## Figures and Tables

**Table 1:** Effects of fasting on lipid profile in overweight and obese population.

**Overweight and Obese Population**								
**-**	**Pre-Fast**				**Post Fast**			
**Article**	**TC**	**TG**	**LDL**	**HDL (mmol/L)**	**TC (mmol/L)**	**TG**	**LDL**	**HDL**
Radhakishun (20)	3.94 mmol/L	0.94 mmol/L	2.40 mmol/L	1.07 mmol/L	4.50 mmol/L	0.94 mmol/L	2.91 mmol/L	1.11 mmol/L
Oh Minsuk (21)	189.8 ± 23.9 mg/dl	108.8 ± 48.7 mg/dl	-	58.5 ± 15.0 mg/dl	193.9 ± 21.6 mg/dl	120.8 ± 80.6 mg/dl	-	61.1 ± 15.4 mg/dl
Bonnet (22)*	-	-	-	-	-	-	-	-
Klempel (23) ADF-HF Group	198 ± 11 mg/dl	123 ± 15 mg/dl	109 ± 9 mg/dl	63 ± 4 mg/dl	172 ± 9 mg/dl	108 ± 15 mg/dl	90 ± 7 mg/dl	63 ± 4 mg/dl
Klempel (23) ADF-LF Group	193 ± 8 mg/dl	97 ± 11 mg/dl	113 ± 7 mg/dl	58 ± 4 mg/dl	162 ± 7 mg/dl	83 ± 10 mg/dl	85 ± 7 mg/dl	60 ± 3 mg/dl

**Note:** *Did not provide pre-fast and post-fast values; only provided the change in LDL and TC.

**Table 2:** Effects of fasting on lipid profile in diabetic population.

**Diabetic Population**								
**-**	**Pre-Fast**				**Post Fast**			
**Article**	**TC**	**TG**	**LDL**	**HDL**	**TC**	**TG**	**LDL**	**HDL**
Bener (24)	4.78 ± 1.02 mmol/L	1.63 ± 0.73 mmol/L	1.88 ± 0.31 mmol/L	1.09 ± 0.27 mmol/L	3.25 ± 1.21 mmol/L	1.52 ± 0.44 mmol/L	2.04 ± 0.86 mmol/L	1.06 ± 0.20 mmol/L
Khan (25)	173.08 ± 36.20 mg/dl	239.95 ± 113.85 mg/dl	104.12 ± 35.51 mg/dl	40.88 ± 9.35 mg/dl	172.27 ± 38.82 mg/dl	207.07 ± 96.44 mg/dl	112.64 ± 34.19 mg/dl	40.34 ± 9.62 mg/dl
Hassanein (10)	158.4 ± 38.1 mmol/L	145.5 ± 72.3 mmol/L	93.3+42.1 mmol/L	49.2 ± 15.7 mmol/L	164.9 ± 47.4 mmol/L	159.3 ± 102.6 mmol/L	97.2+53.9 mmol/L	49.8 ± 14.3 mmol/L

**Table 3:** Effects of fasting on lipid profile in anorexia nervosa population.

**Anorexia Nervosa Population**								
**-**	**Pre-Fast**				**Post Fast**			
**Article**	**TC (mmol/L)**	**TG (mmol/L)**	**LDL (mmol/L)**	**HDL (mmol/L)**	**TC (mmol/L)**	**TG (mmol/L)**	**LDL (mmol/L)**	**HDL (mmol/L)**
Case (26)^	-	-	-	-	5.70 ± 0.76 mmol/L	1.50 ± 1.06 mmol/L	3.40 ± 0.75 mmol/L	1.49 ± 0.26 mmol/L
Giovinazzo* (12)	-	-	-	-	-	-	-	-
-	**Control**				**Anorexia Nervosa**			
-	TC (mmol/L)	TG (mmol/L)	LDL (mmol/L)	HDL (mmol/L)	TC (mmol/L)	TG (mmol/L)	LDL (mmol/L)	HDL (mmol/L)
Weinbrenner (27)**	5.03 ± 0.77	1.0 ± 0.42	3.20 ± 0.71	1.43 ± 0.29	5.48 ± 1.28	0.998 ± 0.44	3.59 ± 1.07	1.53 ± 0.46

**Note:** ^Did not provide baseline data, compared anorexia nervosa group to bulimia group.

*Did not provide pre-fast and post-fast values; only provided that the values increased.

**Provided control versus anorexic population.

**Table 4:** Effects of fasting on lipid profile in healthy population.

**Healthy Population**								
**-**	**Pre-Fast**				**Post Fast**			
**Article**	**TC**	**TG**	**LDL**	**HDL**	**TC**	**TG**	**LDL**	**HDL**
Ziaee V* (28)	168.3 +/- 29.7	66.6 +/- 35.7	115.2 +/- 26.2	40.0 +/- 9.9	170.0 +/- 30.6	69.7 +/- 34.0	119.6 +/- 27.9	36.4 +/- 8.4
Stote (9)**	182.0 ± 8.5 mg/dl	97.1 ± 8.6 mg/dl	109.1 ± 8.6 mg/dl	53.5 ± 3.9 mg/dl	216.5 ± 5.3 mg/dl	93.2 ± 7.7 mg/dl	136.2 ± 4.0 mg/dl	61.9 ± 1.8 mg/dl
Lars (17)	4.90 ± 0.23 mmol/L	-	2.95 ± 0.21 mmol/L	-	6.73 ± 0.41 mm/L	-	4.90 ± 0.36 mmol/L	-
Horne (FEELGOOD Trial) (29,30,31)	-	134 ± 77 mg/dL	104 ± 34 mg/dL	-	-	96 ± 39 mg/dL	123 ± 31 mg/dl	-
Horne (Fasting II Trial) (29,30,31)	-	210 ± 95 mg/dL	92 ± 34 mg/dL	-	-	169 ± 99 mg/dL	101 ± 38 mg/dL	-
Horne (32)	192 ± 27 mg/dl	132 ± 46 mg/dl		54.4 ± 12.1 mg/dl	200 ± 32 mg/dl	94 ± 33 mg/dl	-	57.4 ± 12.1 mg/dl
Mirmiran^ (33)	-	-	-	-	-	-	-	-
Akaberi (34)	172.38 ± 37.62 mg/dl	113.33 ± 49.74 mg/dl	93.21 ± 24.55 mg/dl	33.10 ± 6.53 mg/dl	180.23 ± 31.78 mg/dl	111.87 ± 59.55 mg/dl	98.36 ± 21.94 mg/dl	42.49 ± 8.44 mg/dl
Lamine (35)	3.7 ± 1.0 mmol/L	0.8 ± 0.3 mmol/L	2.3 ± 0.7 mmol/L	1.1 ± 0.4 mmol/L	4.2 ± 1.1 mmol/L	0.7 ± 0.3 mmol/L	2.8 ± 0.9 mmol/L	1.3 ± 0.4 mmol/L

**Note:** *Units were not provided in the study.

**Data for the post-fast is obtained from the participants who consumed 1 meal per day.

^Did not provide pre-fast and post-fast values; only provided that the values increased.

## LIST OF ABBREVIATIONS

ADCR: Alternate Day Caloric Restriction
ADF: Alternate Day Fasting
ApoB: Apolipoprotein B
CR: Calorie Restriction
E-ADCR: Exercise Pulse Alternate Day Caloric Restriction
FFA: Free Fatty Acids
FGF21: Fibroblast Growth Factor 21
GH: Growth Hormone
HDL: High Density Lipoprotein
HF: High Fat
IDL: Intermediate Density Lipoprotein
IF: Intermittent Fasting
IGF-1: Insulin-like Growth Factor 1
LDL: Low Density Lipoprotein
LDL-C: Low-Density Lipid Cholesterol
LF: Low Fat
PCSK9: Proprotein Convertase Subtilisin/Kexin type 9
TC: Total Cholesterol
TG: Triglyceride
VLDL: Very Low-Density Lipoprotein

## STANDARDS OF REPORTING

PRISMA guidelines and methodology were followed.

## SUPPLEMENTARY MATERIAL

PRISMA checklist is available as supplementary material on the publisher’s website along with the published article. Supplementary material is available on the publisher’s website along with the published article.
